# Supplementary material for: HIVprotI: an integrated web based platform for prediction and design of HIV proteins inhibitors
Source: J Cheminform. 2018 Mar 9;10:12. doi: 10.1186/s13321-018-0266-y (PMC5845081; doi:10.1186/s13321-018-0266-y)
Supplement: Supplementary file 2 — Additional file 2. Source code of HIVProtI web server. [file 13321_2018_266_MOESM2_ESM.zip › HIVprotI_Source-code/hivprotI/weka-3-6-6/doc/weka/gui/visualize/Plot2D.html]

Plot2D


---


|  |  |  |
| --- | --- | --- |
| **Overview** \| **Package** \| **Class** \| **Tree** \| **Deprecated** \| **Index** \| **Help** \| **Weka's home** | |  |
| **PREV CLASS**   **NEXT CLASS** | **FRAMES**    **NO FRAMES**     **All Classes** |  |
| SUMMARY: NESTED \| FIELD \| CONSTR \| METHOD | DETAIL: FIELD \| CONSTR \| METHOD |  |


---


## weka.gui.visualize Class Plot2D

```
java.lang.Object
  java.awt.Component
      java.awt.Container
          javax.swing.JComponent
              javax.swing.JPanel
                  weka.gui.visualize.Plot2D
```

**All Implemented Interfaces:**: java.awt.image.ImageObserver, java.awt.MenuContainer, java.io.Serializable, javax.accessibility.Accessible

---

``` public class Plot2D extends javax.swing.JPanel ```

This class plots datasets in two dimensions. It can also plot
classifier errors and clusterer predictions.

**Version:**
:   $Revision: 7059 $

**Author:**
:   Mark Hall (mhall@cs.waikato.ac.nz)

**See Also:**: Serialized Form

---

**Nested Class Summary**

**Nested classes/interfaces inherited from class javax.swing.JComponent**

`javax.swing.JComponent.AccessibleJComponent`

**Nested classes/interfaces inherited from class java.awt.Component**

`java.awt.Component.BaselineResizeBehavior`


| **Field Summary** | |
| --- | --- |
| `static int` | `CONST_AUTOMATIC_SHAPE` |
| `static int` | `DEFAULT_SHAPE_SIZE` |
| `static int` | `DIAMOND_SHAPE` |
| `static int` | `ERROR_SHAPE` |
| `static int` | `MAX_SHAPES` |
| `static int` | `MISSING_SHAPE` |
| `static int` | `PLUS_SHAPE` |
| `static int` | `TRIANGLEDOWN_SHAPE` |
| `static int` | `TRIANGLEUP_SHAPE` |
| `static int` | `X_SHAPE` |

**Fields inherited from class javax.swing.JComponent**

`TOOL_TIP_TEXT_KEY, UNDEFINED_CONDITION, WHEN_ANCESTOR_OF_FOCUSED_COMPONENT, WHEN_FOCUSED, WHEN_IN_FOCUSED_WINDOW`

**Fields inherited from class java.awt.Component**

`BOTTOM_ALIGNMENT, CENTER_ALIGNMENT, LEFT_ALIGNMENT, RIGHT_ALIGNMENT, TOP_ALIGNMENT`

**Fields inherited from interface java.awt.image.ImageObserver**

`ABORT, ALLBITS, ERROR, FRAMEBITS, HEIGHT, PROPERTIES, SOMEBITS, WIDTH`


**Constructor Summary**

`Plot2D()`
  
          Constructor


| **Method Summary** | |
| --- | --- |
| `void` | `addPlot(PlotData2D newPlot)`             Add a plot to the list of plots to display |
| `double` | `convertToAttribX(double scx)`             convert a Panel x coordinate to a raw x value. |
| `double` | `convertToAttribY(double scy)`             convert a Panel y coordinate to a raw y value. |
| `double` | `convertToPanelX(double xval)`             Convert an raw x value to Panel x coordinate. |
| `double` | `convertToPanelY(double yval)`             Convert an raw y value to Panel y coordinate. |
| `void` | `determineBounds()`             Determine the min and max values for axis and colouring attributes |
| `PlotData2D` | `getMasterPlot()`             Get the master plot |
| `double` | `getMaxC()`             Return the current max value of the colouring attribute |
| `double` | `getMaxX()`             Return the current max value of the attribute plotted on the x axis |
| `double` | `getMaxY()`             Return the current max value of the attribute plotted on the y axis |
| `double` | `getMinC()`             Return the current min value of the colouring attribute |
| `double` | `getMinX()`             Return the current min value of the attribute plotted on the x axis |
| `double` | `getMinY()`             Return the current min value of the attribute plotted on the y axis |
| `FastVector` | `getPlots()`             Return the list of plots |
| `static void` | `main(java.lang.String[] args)`             Main method for testing this class |
| `void` | `paintComponent(java.awt.Graphics gx)`             Renders this component |
| `void` | `removeAllPlots()`             Clears all plots |
| `void` | `searchPoints(int x, int y, boolean newFrame)`             Pops up a window displaying attribute information on any instances at a point+-plotting\_point\_size (in panel coordinates) |
| `void` | `setCindex(int c)`             Set the index of the attribute to use for colouring |
| `void` | `setColours(FastVector cols)`             Set a list of colours to use when colouring points according to class values or cluster numbers |
| `void` | `setInstances(Instances inst)`             Sets the master plot from a set of instances |
| `void` | `setJitter(int j)`             Set level of jitter and repaint the plot using the new jitter value |
| `void` | `setMasterPlot(PlotData2D master)`             Set the master plot. |
| `void` | `setPlotCompanion(Plot2DCompanion p)`             Set a companion class. |
| `void` | `setXindex(int x)`             Set the index of the attribute to go on the x axis |
| `void` | `setYindex(int y)`             Set the index of the attribute to go on the y axis |

**Methods inherited from class javax.swing.JPanel**

`getAccessibleContext, getUI, getUIClassID, setUI, updateUI`

**Methods inherited from class javax.swing.JComponent**

`addAncestorListener, addNotify, addVetoableChangeListener, computeVisibleRect, contains, createToolTip, disable, enable, firePropertyChange, firePropertyChange, firePropertyChange, getActionForKeyStroke, getActionMap, getAlignmentX, getAlignmentY, getAncestorListeners, getAutoscrolls, getBaseline, getBaselineResizeBehavior, getBorder, getBounds, getClientProperty, getComponentPopupMenu, getConditionForKeyStroke, getDebugGraphicsOptions, getDefaultLocale, getFontMetrics, getGraphics, getHeight, getInheritsPopupMenu, getInputMap, getInputMap, getInputVerifier, getInsets, getInsets, getListeners, getLocation, getMaximumSize, getMinimumSize, getNextFocusableComponent, getPopupLocation, getPreferredSize, getRegisteredKeyStrokes, getRootPane, getSize, getToolTipLocation, getToolTipText, getToolTipText, getTopLevelAncestor, getTransferHandler, getVerifyInputWhenFocusTarget, getVetoableChangeListeners, getVisibleRect, getWidth, getX, getY, grabFocus, isDoubleBuffered, isLightweightComponent, isManagingFocus, isOpaque, isOptimizedDrawingEnabled, isPaintingForPrint, isPaintingTile, isRequestFocusEnabled, isValidateRoot, paint, paintImmediately, paintImmediately, print, printAll, putClientProperty, registerKeyboardAction, registerKeyboardAction, removeAncestorListener, removeNotify, removeVetoableChangeListener, repaint, repaint, requestDefaultFocus, requestFocus, requestFocus, requestFocusInWindow, resetKeyboardActions, reshape, revalidate, scrollRectToVisible, setActionMap, setAlignmentX, setAlignmentY, setAutoscrolls, setBackground, setBorder, setComponentPopupMenu, setDebugGraphicsOptions, setDefaultLocale, setDoubleBuffered, setEnabled, setFocusTraversalKeys, setFont, setForeground, setInheritsPopupMenu, setInputMap, setInputVerifier, setMaximumSize, setMinimumSize, setNextFocusableComponent, setOpaque, setPreferredSize, setRequestFocusEnabled, setToolTipText, setTransferHandler, setVerifyInputWhenFocusTarget, setVisible, unregisterKeyboardAction, update`

**Methods inherited from class java.awt.Container**

`add, add, add, add, add, addContainerListener, addPropertyChangeListener, addPropertyChangeListener, applyComponentOrientation, areFocusTraversalKeysSet, countComponents, deliverEvent, doLayout, findComponentAt, findComponentAt, getComponent, getComponentAt, getComponentAt, getComponentCount, getComponents, getComponentZOrder, getContainerListeners, getFocusTraversalKeys, getFocusTraversalPolicy, getLayout, getMousePosition, insets, invalidate, isAncestorOf, isFocusCycleRoot, isFocusCycleRoot, isFocusTraversalPolicyProvider, isFocusTraversalPolicySet, layout, list, list, locate, minimumSize, paintComponents, preferredSize, printComponents, remove, remove, removeAll, removeContainerListener, setComponentZOrder, setFocusCycleRoot, setFocusTraversalPolicy, setFocusTraversalPolicyProvider, setLayout, transferFocusBackward, transferFocusDownCycle, validate`

**Methods inherited from class java.awt.Component**

`action, add, addComponentListener, addFocusListener, addHierarchyBoundsListener, addHierarchyListener, addInputMethodListener, addKeyListener, addMouseListener, addMouseMotionListener, addMouseWheelListener, bounds, checkImage, checkImage, contains, createImage, createImage, createVolatileImage, createVolatileImage, dispatchEvent, enable, enableInputMethods, firePropertyChange, firePropertyChange, firePropertyChange, firePropertyChange, firePropertyChange, getBackground, getBounds, getColorModel, getComponentListeners, getComponentOrientation, getCursor, getDropTarget, getFocusCycleRootAncestor, getFocusListeners, getFocusTraversalKeysEnabled, getFont, getForeground, getGraphicsConfiguration, getHierarchyBoundsListeners, getHierarchyListeners, getIgnoreRepaint, getInputContext, getInputMethodListeners, getInputMethodRequests, getKeyListeners, getLocale, getLocation, getLocationOnScreen, getMouseListeners, getMouseMotionListeners, getMousePosition, getMouseWheelListeners, getName, getParent, getPeer, getPropertyChangeListeners, getPropertyChangeListeners, getSize, getToolkit, getTreeLock, gotFocus, handleEvent, hasFocus, hide, imageUpdate, inside, isBackgroundSet, isCursorSet, isDisplayable, isEnabled, isFocusable, isFocusOwner, isFocusTraversable, isFontSet, isForegroundSet, isLightweight, isMaximumSizeSet, isMinimumSizeSet, isPreferredSizeSet, isShowing, isValid, isVisible, keyDown, keyUp, list, list, list, location, lostFocus, mouseDown, mouseDrag, mouseEnter, mouseExit, mouseMove, mouseUp, move, nextFocus, paintAll, postEvent, prepareImage, prepareImage, remove, removeComponentListener, removeFocusListener, removeHierarchyBoundsListener, removeHierarchyListener, removeInputMethodListener, removeKeyListener, removeMouseListener, removeMouseMotionListener, removeMouseWheelListener, removePropertyChangeListener, removePropertyChangeListener, repaint, repaint, repaint, resize, resize, setBounds, setBounds, setComponentOrientation, setCursor, setDropTarget, setFocusable, setFocusTraversalKeysEnabled, setIgnoreRepaint, setLocale, setLocation, setLocation, setName, setSize, setSize, show, show, size, toString, transferFocus, transferFocusUpCycle`

**Methods inherited from class java.lang.Object**

`equals, getClass, hashCode, notify, notifyAll, wait, wait, wait`

**Field Detail**

### MAX\_SHAPES

```
public static final int MAX_SHAPES
```

**See Also:**: Constant Field Values

---


### ERROR\_SHAPE

```
public static final int ERROR_SHAPE
```

**See Also:**: Constant Field Values

---


### MISSING\_SHAPE

```
public static final int MISSING_SHAPE
```

**See Also:**: Constant Field Values

---


### CONST\_AUTOMATIC\_SHAPE

```
public static final int CONST_AUTOMATIC_SHAPE
```

**See Also:**: Constant Field Values

---


### X\_SHAPE

```
public static final int X_SHAPE
```

**See Also:**: Constant Field Values

---


### PLUS\_SHAPE

```
public static final int PLUS_SHAPE
```

**See Also:**: Constant Field Values

---


### DIAMOND\_SHAPE

```
public static final int DIAMOND_SHAPE
```

**See Also:**: Constant Field Values

---


### TRIANGLEUP\_SHAPE

```
public static final int TRIANGLEUP_SHAPE
```

**See Also:**: Constant Field Values

---


### TRIANGLEDOWN\_SHAPE

```
public static final int TRIANGLEDOWN_SHAPE
```

**See Also:**: Constant Field Values

---


### DEFAULT\_SHAPE\_SIZE

```
public static final int DEFAULT_SHAPE_SIZE
```

**See Also:**: Constant Field Values


**Constructor Detail**

### Plot2D

```
public Plot2D()
```

:   Constructor


**Method Detail**

### setPlotCompanion

```
public void setPlotCompanion(Plot2DCompanion p)
```

:   Set a companion class. This is a class that might want
    to render something on the plot before we do our thing. Eg,
    Malcolm's shape drawing stuff needs to happen before we plot
    axis and points

    :   **Parameters:**: `p` - a companion class

---


### setJitter

```
public void setJitter(int j)
```

:   Set level of jitter and repaint the plot using the new jitter value

    :   **Parameters:**: `j` - the level of jitter

---


### setColours

```
public void setColours(FastVector cols)
```

:   Set a list of colours to use when colouring points according
    to class values or cluster numbers

    :   **Parameters:**: `cols` - the list of colours to use

---


### setXindex

```
public void setXindex(int x)
```

:   Set the index of the attribute to go on the x axis

    :   **Parameters:**: `x` - the index of the attribute to use on the x axis

---


### setYindex

```
public void setYindex(int y)
```

:   Set the index of the attribute to go on the y axis

    :   **Parameters:**: `y` - the index of the attribute to use on the y axis

---


### setCindex

```
public void setCindex(int c)
```

:   Set the index of the attribute to use for colouring

    :   **Parameters:**: `c` - the index of the attribute to use for colouring

---


### getPlots

```
public FastVector getPlots()
```

:   Return the list of plots

    :   **Returns:**: the list of plots

---


### getMasterPlot

```
public PlotData2D getMasterPlot()
```

:   Get the master plot

    :   **Returns:**: the master plot

---


### getMaxX

```
public double getMaxX()
```

:   Return the current max value of the attribute plotted on the x axis

    :   **Returns:**: the max x value

---


### getMaxY

```
public double getMaxY()
```

:   Return the current max value of the attribute plotted on the y axis

    :   **Returns:**: the max y value

---


### getMinX

```
public double getMinX()
```

:   Return the current min value of the attribute plotted on the x axis

    :   **Returns:**: the min x value

---


### getMinY

```
public double getMinY()
```

:   Return the current min value of the attribute plotted on the y axis

    :   **Returns:**: the min y value

---


### getMaxC

```
public double getMaxC()
```

:   Return the current max value of the colouring attribute

    :   **Returns:**: the max colour value

---


### getMinC

```
public double getMinC()
```

:   Return the current min value of the colouring attribute

    :   **Returns:**: the min colour value

---


### setInstances

```
public void setInstances(Instances inst)
                  throws java.lang.Exception
```

:   Sets the master plot from a set of instances

    :   **Parameters:**: `inst` - the instances **Throws:**: `java.lang.Exception` - if instances could not be set

---


### setMasterPlot

```
public void setMasterPlot(PlotData2D master)
                   throws java.lang.Exception
```

:   Set the master plot.

    :   **Parameters:**: `master` - the plot to make the master plot **Throws:**: `java.lang.Exception` - if the plot could not be set.

---


### removeAllPlots

```
public void removeAllPlots()
```

:   Clears all plots

---


### addPlot

```
public void addPlot(PlotData2D newPlot)
             throws java.lang.Exception
```

:   Add a plot to the list of plots to display

    :   **Parameters:**: `newPlot` - the new plot to add **Throws:**: `java.lang.Exception` - if the plot could not be added

---


### searchPoints

```
public void searchPoints(int x,
                         int y,
                         boolean newFrame)
```

:   Pops up a window displaying attribute information on any instances
    at a point+-plotting\_point\_size (in panel coordinates)

    :   **Parameters:**: `x` - the x value of the clicked point: `y` - the y value of the clicked point: `newFrame` - true if instance info is to be displayed in a new frame.

---


### determineBounds

```
public void determineBounds()
```

:   Determine the min and max values for axis and colouring attributes

---


### convertToAttribX

```
public double convertToAttribX(double scx)
```

:   convert a Panel x coordinate to a raw x value.

    :   **Parameters:**: `scx` - The Panel x coordinate **Returns:**: A raw x value.

---


### convertToAttribY

```
public double convertToAttribY(double scy)
```

:   convert a Panel y coordinate to a raw y value.

    :   **Parameters:**: `scy` - The Panel y coordinate **Returns:**: A raw y value.

---


### convertToPanelX

```
public double convertToPanelX(double xval)
```

:   Convert an raw x value to Panel x coordinate.

    :   **Parameters:**: `xval` - the raw x value **Returns:**: an x value for plotting in the panel.

---


### convertToPanelY

```
public double convertToPanelY(double yval)
```

:   Convert an raw y value to Panel y coordinate.

    :   **Parameters:**: `yval` - the raw y value **Returns:**: an y value for plotting in the panel.

---


### paintComponent

```
public void paintComponent(java.awt.Graphics gx)
```

:   Renders this component

    :   **Overrides:**: `paintComponent` in class `javax.swing.JComponent`
    :   **Parameters:**: `gx` - the graphics context

---


### main

```
public static void main(java.lang.String[] args)
```

:   Main method for testing this class

    :   **Parameters:**: `args` - arguments


---


|  |  |  |
| --- | --- | --- |
| **Overview** \| **Package** \| **Class** \| **Tree** \| **Deprecated** \| **Index** \| **Help** \| **Weka's home** | |  |
| **PREV CLASS**   **NEXT CLASS** | **FRAMES**    **NO FRAMES**     **All Classes** |  |
| SUMMARY: NESTED \| FIELD \| CONSTR \| METHOD | DETAIL: FIELD \| CONSTR \| METHOD |  |


---
